# Counteracting muscle wasting in aging and neuromuscular diseases: the critical role of IGF-1

**DOI:** 10.18632/aging.100050

**Published:** 2009-05-13

**Authors:** Bianca Maria Scicchitano, Emanuele Rizzuto, Antonio Musarò

**Affiliations:** Institute Pasteur Cenci-Bolognetti, Department of Histology and Medical Embryology, IIM, Sapienza University of Rome, Rome 00161, Italy

**Keywords:** Aging, muscle wasting, IGF-1, neuromuscular diseases, ALS, muscular dystrophy

## Abstract

Most muscle pathologies are characterized by the progressive loss of muscle tissue due to chronic degeneration combined with the inability of regeneration machinery to replace the damaged muscle. These pathological changes, known as muscle wasting, can be attributed to the activation of several proteolytic systems, such as calpain, ubiquitin-proteasome and caspases, and to the alteration in muscle growth factors. Among them, insulin-like growth factor-1 (IGF-1) has been implicated in the control of skeletal muscle growth, differentiation, survival, and regeneration and has been considered a promising therapeutic agent in staving off the advance of muscle weakness. Here we review the molecular basis of muscle wasting associated with diseases, such as sarcopenia, muscular dystrophy and Amyotrophic Lateral Sclerosis, and discuss the potential therapeutic role of local IGF-1 isoforms in muscle aging and diseases.

## Introduction

It is generally accepted that the primary cause of functional impairment in muscle is a cumulative failure to repair damage related to an overall decrease in anabolic processes. Despite numerous theories and intensive research, the principal molecular mechanisms underlying the process of muscle wasting are still unknown.

Current data point out that muscle wasting is a multifactorial process and believed to be the result of both intrinsic factors, involving changes in molecular and cellular levels, and extrinsic ones, such as nutrition and exercise [1]. Among intrinsic factors, the proteolytic systems have been postulated to be responsible for the protein breakdown.   Calpain-,  ubiquitin- and caspase- mediated protein degradation are the principal proteolytic pathways activated in several pathologies, leading to myofiber degeneration, and impaired muscle regeneration.

Calpains are calcium-activated cysteine proteases that participate in various intracellular signal transduction pathways mediated by Ca^2+^ [2], causing disruption of the contractile tissue, mitochondrial swelling, sarcoplasmic reticulum vacuolization, and sarcomeric alterations.

The ubiquitin-proteasome pathway plays a key role in the turnover of muscle protein and the pathway involves an enzymatic cascade starting with the ubiquitination of muscle protein to be degraded by the 26S proteasome in a process that unfolds the protein, releases ubiquitin, and degrades the protein to small peptides and amino acids [3].

Caspases are a family of cysteine proteases, representing central components of the apoptotic machinery in several tissues [4].

Additionally, many other factors, including stress oxidative damage and alteration in satellite cells activity may all contribute to muscle wasting [5,6].

In designing therapies that can counteract muscle wasting it is important to choose molecules able to maintain muscle mass, suppress muscle loss and stimulate muscle regeneration. In this context, one of the potential candidates is the insulin-like growth factor-1 (IGF-1), involved in several anabolic process in skeletal muscle [7].

### The molecular complexities of IGF-1 transcription

**Figure 1.**
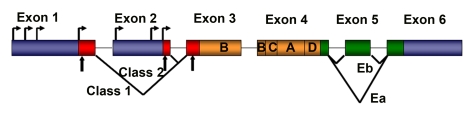
Schematic representation of rodent IGF-1 gene. The rodent IGF-1 gene contains six exons (colored boxes), separated by five introns (black lines). Both exons 1 and 2 contain multiple transcription start sites (horizontal arrows). Translation initiation codons (AUG) are located at exons 1, 2 and 3 (vertical arrows). Exons 1, 2 and 3 code for the signal peptide of precursor IGF-1 (red boxes). Exons 5 and 6 each encode distinct portions of the E-peptides (green boxes).

An impressive body of knowledge has been accumulated since the IGF-1 locus was first described, but surprisingly the potential diversity of roles played by different IGF-1 isoforms has only recently been appreciated. As its name implies, IGF-1 is similar to insulin in structure, with it shares a 50% amino acid identity. However, unlike the insulin gene, the single-copy IGF-1 gene locus encodes multiple proteins with variable amino- and carboxy-terminal amino acid sequences (Figure 1). The amino acid sequence of the mature peptide differs from that of insulin by retention of the C peptide, by a short extension of the A chain to include a novel domain D, and by the presence of variable C-terminal E peptides.

Although the IGF-1 gene is highly conserved in numerous species, its relatively large size (>70 kb), and its complex transcriptional and splicing pattern, have complicated its analysis.

The rodent IGF-1 gene contains six exons, separated by five introns (Figure 1) [8]. Exons 1 and 2 encode distinct 5′UTRs, as well as different parts of the signal peptide, and are therefore termed leader exons. Exon 3 encodes 27 amino acids that are part of the signal peptide and common to all isoforms, as well as part of the mature IGF-1 peptide.

Exon 4 encodes the rest of the mature peptide and 16 amino acids of the amino-terminal region of the E-peptide, which is also common to all IGF-1 mRNAs. Exons 5 and 6 encode two distinct carboxy-terminal E-peptides and the 3′UTR.

Although IGF-1 transcripts are not exclusively tissue-restricted, those that initiate at Exon 2 predominate in the liver, are highly growth hormone responsive and as such are major endocrine effectors of GH [9]. By contrast, transcripts initiating at Exon 1 are widely expressed in all tissues, and are less effected by circulating growth hormone levels, presumably performing autocrine or paracrine functions. The alternate splicing at the 5' ends of these two IGF-1 transcripts generates different signal peptides, which purportedly affects the precise N-terminal pro-peptide cleavage site [9]. The function of the proteins encoded by these different transcripts is widely debated but a cohesive picture has yet to emerge [10].

Elucidation of isoform function is also complicated by alternate splicing at the 3' end of IGF-1 transcripts. This produces variability in the length and amino acid sequence of the E peptide, and in the length and base sequence of the 3'UTR. To date, two different splice patterns have been documented in rodents (Figure 1). Each generates E peptides with a common N-terminal 16 aa sequence, and alternate C-terminal sequences [8,11]. If Exon 4 splices to Exon 6 (the predominant pattern), the length of the 3'UTR is highly variable, but in all cases the Ea peptide is generated with 19 additional amino acids. If Exon 4 splices to Exon 5 and 6, a variant known as Eb is encoded, which is frameshifted relative to Exon 6 and therefore a different 25 aa sequence is added to the common 16 aa encoded by Exon 4.

Although E peptide choice appears to be independent of promoter use, Eb-containing transcripts are more abundant in liver, whereas Ea-containing transcripts are widespread in extra-hepatic tissues. In addition, the analysis of the amino acid structure of both E-peptides has revealed the presence of two N-linked glycosylation sites only in the Ea peptide, but not in the Eb peptide, suggesting that this post-translational modification is involved in a biological action of the IGF-1 isoform [11].

The IGF-1Eb isoform is also up-regulated in muscles subjected to stretch and has been named mechano growth factor (MGF) [12]. The determination of E peptide function and fate awaits the availability of epitope-specific antibodies, since it is unclear when or whether E peptides are cleaved from the mature IGF-1 protein. Notably, E peptide splicing patterns are different in the human gene [8], an anomaly that will need to be considered in the future when translating the results of animal research into clinical applications.

### The importance of IGF-1 isoforms

Analyses of transgenic mice expressing different IGF-1 isoforms have provided insight into the role of IGF-1 signaling in the physiology of striated muscle [7]. The fact that IGF-1 can act either as a circulating hormone or as a local growth factor has confounded previous analyses of animal models in which transgenic IGF-1 synthesized in extra-hepatic tissues was released into the circulation. Thus, over-expression of one IGF-1 isoform in the heart prevented activation of cell death in the viable myocardium after infarction, limiting ventricular dilation, myocardial loading, cardiac hypertrophy, and diabetic cardiomyopathy, supporting the notion that constitutive over-expression of IGF-1 in cardiomyocytes protects them from apoptosis and hypertrophy in the normal and pathological heart [13,14]. However, in another study, over-expression of a different IGF-1-transgene in the heart induced physiological cardiac hypertrophy that progressed to maladaptive hypertrophy [15]. The discrepancies in these phenotypes underscore the normal physiological difference between IGF-1 isoform function. In addition, substantial evidence supports the involvement of IGF-1 in mitogenesis and neoplastic transformation [16], suggesting that this signaling pathway plays an important role in the process of tumor promotion. The neoplastic potential of at least certain IGF-1 isoforms is an obvious concern to be taken into account when designing IGF-therapeutic strategies for human pathologies, where the specific role of each IGF-1 isoform must be viewed in the appropriate tissue context.

Thus, restricting the action of supplemental IGF-1 to the tissue of origin by use of a local IGF-1 isoform will allow the assessment of its autocrine/paracrine role in skeletal muscle throughout the life-span of the animal, exclusive of possible endocrine effects on other tissues.

### The effects of local isoform of IGF-1 on muscle homeostasis

### mIGF-1 and muscle aging

The prolongation of skeletal muscle strength in aging and neuromuscular disease has been the objective of numerous studies employing a variety of approaches.

IGF-1, involved in muscle growth and hypertrophy, decline during postnatal life, raising the prospect that this decline contributes to the progress of muscle atrophy in senescence, and limits the ability of skeletal muscle tissue to effect repair or to regenerate.

To test this possibility we generated a transgenic mouse in which the local isoform of IGF-1 (mIGF-1) is driven by MLC promoter (MLC/mIGF-1) [17]. The MLC regulatory elements included in this construct activate linked gene expression as early as E9.5 days in embryonic mouse development, and expression continues to be high in the fastest Type IIb fibers. Transgenic animals exhibits marked skeletal muscle hypertrophy with no undesirable side effects such as tumor formation.

**Figure 2.**
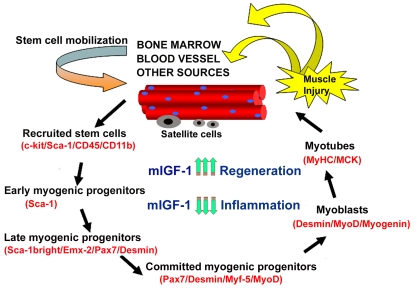
Model of stem cell-mediated muscle regeneration. (modified from ref. 18). Muscle injury involves the activation of satellite cells and the recruitment of circulating stem cells, which when penetrating the muscle compartment receive myogenic signals and may contribute to muscle regeneration and repair. This process is enhanced by mIGF-1 expression. By modulating the inflammatory response and reducing fibrosis, supplemental mIGF-1 creates a qualitatively different environment for sustaining more efficient muscle regeneration and repair.

The increased muscle mass in mIGF-1 transgenic mice was associated with augmented force generation compared to age-matched wild type littermates [17]. Examination of two year-old animals revealed that whereas wild type mice underwent characteristic muscle atrophy, expression of the mIGF-1 transgene was protective against normal loss of muscle mass during senescence [17]. Over-expression of the mIGF-1 transgene also preserved the regenerative capacity of senescent muscle tissues stimulating both the activity of satellite cells and the recruitment of circulating stem cells [17,18] (Figure 2). We demonstrated that upon muscle injury, stem cells expressing c-Kit, Sca-1, and CD45 antigens increased locally and the percentage of the recruited cells were conspicuously enhanced by mIGF-1 expression [18]. More recently, we demonstrated that local expression of mIGF-1 accelerates the regenerative process of injured skeletal muscle, negatively modulating the inflammatory response [19]. These data indicate that  mIGF-1 promote a qualitative environment, guaranteeing a more efficient muscle regeneration process. Thus mIGF-1 can overcome the normal inability of skeletal muscle to sustain regeneration and repair and as such represents a potentially effective gene therapeutic strategy to combat muscle wasting.   This hypothesis was supported by the demonstration that the action of mIGF-1 is not dependent on life-long expression. Introduction of mIGF-1 somatically using an Adeno-Associated-Viral (AAV) vector was sufficient to rejuvenate the leg muscles of 27 month old mice, which exhibited the same mechanical force as legs of younger mice, and did not develop the pathological characteristics of senescent muscle [20].

The importance of appropriate IGF-1 isoform selection is further underscored by preliminary analysis of mouse lines generated with a second IGF-1 transgene (cIGF-1), which differs from the mIGF-1 only in a variant C-terminal peptide. These animals did not display pronounced muscle hypertrophy but had increased levels of circulating IGF-1, mild cardiac hypertrophy, an increased incidence of late onset neoplasia (unpublished observation). Thus, the choice of isoform is critical to the design of gene therapeutic strategies employing IGF-1.

### mIGF-1 and muscular dystrophy

Muscular dystrophies are degenerative disorders characterized by progressive weakness in specific muscle groups, persistent protein degradation and alteration in the regenerative capacity of muscle satellite cells [21]. Mutations in genes encoding proteins of the dystrophin-glycoprotein complex (DGC) lead to alteration in muscle structure and cause muscular dystrophy [21,22]. Without dystrophin, the DGC is unstable leading to an increase in muscle damage. Different studies support the notion that loss of the link between extracellular matrix and cytoskeleton represents the critical parameter for the maintenance of the structural integrity of skeletal muscle [23].

A further complication that exacerbates muscular dystrophy is the persistence of inflammation. In normal skeletal muscle, damage is followed by an inflammatory response [24] involving multiple cell types that subsides after several days. This transient inflammatory response is a normal homeostatic reaction to myonecrosis and is necessary for efficient repair. However a persistent inflammatory response is observed in dystrophic muscle, leading to an altered extracellular environment [25], including an increased presence of inflammatory cells (e.g., macrophages) and elevated levels of various inflammatory cytokines (e.g., TNF-alpha, TGF-beta).

Because it is clear that mIGF-1 can prevent aging- related loss of muscle function, stimulates muscle regeneration and modulates the inflammatory response in damaged muscle, it is possible that mIGF-1 can prevent or diminish muscle loss associated with diseases.

To prove this hypothesis, we introduced mIGF-1 into the mdx dystrophic animals (mdx/mIGF-1) [26]. By analyzing muscle morphology and function in transgenic mdx/mIGF-1 mice we observed significant improvement in muscle mass and strength, a decrease in myonecrosis, and a reduction in fibrosis in aged diaphragms [26]. In particular, even though IGF-1 has been shown to stimulate fibroblasts, there was a net decrease in fibrosis in diaphragms of the mdx/mIGF-1 mice [26]. It may be that the efficient and rapid repair of the mdx/mIGF-1 muscles prevents the establishment of an environment into which the fibroblasts migrate. This is of particular relevance to the human dystrophic condition where virtually all skeletal muscles succumb to fibrosis.

Finally, signaling pathways associated with muscle regeneration and protection against apoptosis were significantly elevated [26]. These results suggest that a combination of promoting muscle regenerative capacity and preventing muscle necrosis could be an effective treatment for the secondary symptoms caused by the primary loss of dystrophin.

In addition, another study demonstrated that coinjection of the rAAV-microdystrophin and rAAV-mIGF-1 vectors resulted in increased muscle mass and strength, reduced myofiber degeneration, and increased protection against contraction-induced injury [27]. These results suggest that a dual-gene combinatorial strategy could enhance the efficacy of gene therapy of DMD and underscored the importance of rAAV vectors due to their relative lack of immunologic and toxic side effect combined with their potential for body-wide systemic gene delivery to muscle [27].

### mIGF-1 and amyotrophic lateral sclerosis (ALS)

ALS is a progressive, lethal neuromuscular disease associated with the degeneration of motor neurons, leading to muscle atrophy and paralysis [28]. Although a significant proportion of familial ALS results from a toxic gain-of-function associated with dominant SOD1 mutations, the etiology of the disease and its specific cellular origins have remained difficult to define.

Notably, restriction of SOD1 mutant expression selectively to post-natal motor neurons failed to produce detectable sign of pathology or motor-neuron disease [29], suggesting that other cell types may be involved in ALS-associated neurodegeneration. Indeed, analysis of chimeras generated between wild type and SOD1 mutant mouse embryonic cells revealed that wild type non neuronal cells in adult chimeric animals extended the survival of SOD1 mutant motor neurons, suggesting that the neurodegenerative action of mutant SOD1 may operate through a dominant paracrine activity emanating from non neuronal cells [30].

Skeletal muscle is an untested component in the motor neurodegenerative effects of SOD1 mutations. More recently, we addressed this critical aspect of the pathogenesis of ALS, demonstrating that skeletal muscle is a direct target of SOD1^G93A^-mediated toxicity [31], refocusing therapeutic strategies to attenuate motor neuronal degradation towards skeletal muscle.

Adult muscle fibers are a source of signals that influence neuron survival, axonal growth and maintenance of synaptic connections. Among them IGF-1 has also been implicated in anabolism of nerve tissue, promoting neuronal survival [7].

Recently, the potential beneficial effect of human recombinant IGF-1 on ALS patients has been tested, however the results were doubtful [32]. In particular, the subcutaneously injection of IGF-1 did not show beneficial effects in ALS patients [32]. The critical problem could be the failure to deliver the neurothophin effectively to the target cells and tissue. Moreover, the IGF-1 system, as discussed above, is complex, since multiple transcripts of the IGF-1 gene encode different isoforms, which induce different cellular responses. This hypothesis was supported by the evidences that either AAV-mIGF-1 mediated muscle delivery [33] or localized expression of co-inherited MLC/mIGF-1 transgene exclusively in the muscles of SOD1^G93A^ mouse [34,35] counteracts the symptoms of ALS and reduces components of catabolism, activating satellite cell and markers of regeneration [33-35]. The protective effects of muscle-restricted mIGF-1 against the dominant action of mutant SOD1^G93A^ stabilized also neuromuscular junctions and led to a reduction in astrocytosis/inflammation in the spinal cord, enhancing motor neuronal survival.

## Conclusions

These preliminary studies provide exciting avenues for future discovery, however true innovation in this field will undoubtedly derive from the integration of our insights with other key advances in regenerative research, to form a cohesive and coherent strategy that addresses the short, medium and long-term aspects of the therapeutic process.
